# Iodide of Potassium in Potts’ Paraplegia

**Published:** 1893-06

**Authors:** Samuel E. Milliken

**Affiliations:** New York; Lecturer on Surgery in the New York Polyclinic; 157 Madison Avenue


### IODIDE OF POTASSIUM IN POTTS' PARAPLEGIA.

By SAMUEL E. MILLIKEN, M. D., of New York.
Lecturer on Surgery in the New York Polyclinic.

The paralysis following Potts' disease of the spine is a well-known complication which occurs most frequently when the cervical or upper dorsal region is involved. The general supposition that the paralysis is due to the angular deformity is wrong, as is not only demonstrated by post-mortem examinations, but by the clinical history of the disease. On the contrary, it is due to the pachymeningitis and the infiltration of the surrounding structures which produce compression of the spinal cord. The fact that paralysis occurs only in the course of the trouble when only slight deformity exists, together with a natural tendency toward recovery in cases with great deformity, go to prove the assertion above made. The duration of paralysis is from six to eighteen months where careful mechanical treatment is carried out, but can be much shortened by the administration of increasing doses of potassium iodide.

It should be begun with ten drops of the saturated solution, one or two hours after meals, and rapidly increased to fifty or sixty drops three times a day. Milk is the best medium for administration, but many cases will not require other than a thorough solution with water.

The patient should be kept in the recumbent posture, with perfect fixation of the spinal column by means of a brace or plaster-of-Paris jacket. When the disease is located in the upper dorsal or cervical region, it is well to keep up continuous extension by means of a band around the head or a jury-mast apparatus from the chin and occiput.

Three or four pounds will usually suffice with children, and the weight required to be less at night than during the day.

The mortality from laminectomy is large and it should not be performed until the spine has been subjected to careful mechanical support, with the patient in the recumbent posture and a thorough course of potassium iodide.

157 MADISON AVENUE.

---

RHEUMATISM:

| | | |
|---|---|---|
| ℞—Pot. Iodide | 2½ | drachms, |
| Tinct. Cimicifuga | 1½ | ounces. |
| Vin. Colch. sem | 1 | ounce. |
| Fl. Ext. Henbane | ½ | ounce. |
| Simple Syrup | 5 | ounces. |

M. Sig. Teaspoonful, well diluted with water, every four hours.

